# Successful non-surgical management of locally advanced gastric adenocarcinoma with squamous differentiation and programmed death-ligand 1 combined positive score 75: a case report

**DOI:** 10.1186/s13256-026-06440-x

**Published:** 2026-07-31

**Authors:** Sandy Ghadeer Jalloul, Joudi Ghadeer Jalloul, Sarah Ghadeer Jalloul

**Affiliations:** Al Andalus University for Medical Sciences, Tartus, Syria

**Keywords:** Gastric adenocarcinoma, Poorly differentiated carcinoma, Squamous differentiation, PD-L1 expression, Chemotherapy, Immunotherapy, Nivolumab, Non-surgical intervention, Case report

## Abstract

**Background:**

Poorly differentiated gastric adenocarcinoma with squamous differentiation is a rare and aggressive malignancy, with limited representation in the literature. Its biological behaviour remains poorly characterized, and prognosis is generally poor. Here, we present a case that highlights the role of biomarker-driven, non-surgical management in advanced gastric malignancy, particularly in the context of high PD-L1 expression in an elderly patient.

**Case presentation:**

A 75-year-old Syrian Arab male presented with anaemia, weight loss and anorexia. Imaging revealed a large ulcerated gastric mass measuring 9 × 11 cm with perigastric lymphadenopathy and local invasion, but no evidence of distant metastasis. Histopathological examination confirmed poorly differentiated adenocarcinoma with squamous differentiation. Immunohistochemistry demonstrated diffuse p40 positivity and focal cytokeratin 7 (CK7) positivity. Notably, programmed death-ligand 1 (PD-L1) expression was strongly positive, with a combined positive score (CPS) of 75. The patient was started on combined chemotherapy (oxaliplatin and 5-fluorouracil) and immunotherapy (nivolumab). Remarkably, follow-up positron emission tomography–computed tomography (PET-CT) at 3 months showed a reduction in tumour size and metabolic activity, with near complete radiological remission by 6 months. At 1-year follow-up, the patient remains clinically well and disease-free without surgical intervention.

**Conclusions:**

This case illustrates how high PD-L1 expression may substantially alter the expected clinical course of gastric cancer, even in rare and poorly differentiated subtypes typically associated with poor outcomes. The observed durable clinical complete response underscores the potential of biomarker-guided chemo-immunotherapy and supports consideration of non-surgical approaches in selected patients.

## Background

Gastric cancer remains a leading cause of cancer-related mortality worldwide [1], with poorly differentiated adenocarcinomas representing an aggressive subtype associated with a poor prognosis [2].

While gastric adenocarcinoma (GAC) is common, the presence of squamous differentiation is rare, occurring in less than 2% of gastric malignancies [3]. The pathogenesis and clinical significance of this differentiation remain poorly understood due to the limited number of cases described in the literature [3].

A definitive diagnosis of adenosquamous carcinoma (ASC) requires a neoplastic squamous component of at least 25% of the total tumour volume [4]; cases falling below this threshold are classified as adenocarcinoma with squamous differentiation.

Notably, PD-L1 [5] expression has been reported in approximately 55–66% of advanced gastric cancers. The combined positive score (CPS) is the established biomarker for predicting immunotherapy response. Pivotal phase III trials, such as CheckMate 649, demonstrated that patients with a CPS ≥ 5 achieve significant survival benefits when nivolumab is added to standard chemotherapy in the first-line advanced or metastatic setting [6]. Although clinical success with this regimen is mostly reported in the context of perioperative or palliative settings, achieving a durable radiological and metabolic complete response through exclusive systemic therapy in locally advanced, non-metastatic disease remains an area of intense investigation [6].

We report a rare case of exceptionally high PD-L1-expressing (CPS 75) poorly differentiated gastric adenocarcinoma with squamous differentiation that achieved long-term remission without surgical intervention.

What makes this case particularly noteworthy is that: despite multiple aggressive pathological features usually associated with poor prognosis—including poor differentiation, high grade dysplasia, and squamous differentiation—in an elderly patient with comorbidities [7], within 1 year of treatment, the patient exhibited sustained disease control without the need for surgical intervention. This case contributes to the limited body of literature on this unusual histopathological entity and supports further research into the organ-sparing potential of immunotherapy in super-responders.

## Case presentation

A 75-year-old Syrian Arab male was referred for evaluation of persistent anaemia, unintentional weight loss, and decreased appetite. The patient’s past medical history was notable for diabetes mellitus treated with antidiabetic medication, hypertension managed with antihypertensive therapy and a recent diagnosis of atrial fibrillation. He was a former smoker and reported occasional alcohol consumption. He is currently retired and lives with his wife. Family history was notable for gastric cancer in his mother and gastric ulcer in his father.

Prior to referral, the patient had already undergone a course of oral antibiotics for prostatitis, following which clinical signs of inflammation resolved and prostate-specific antigen (PSA) levels normalized. Despite this, the anaemia and systemic symptoms persisted, prompting referral to a gastroenterologist for further investigation. On physical examination, the patient appeared stable and in no acute distress, with a baseline Eastern Cooperative Oncology Group (ECOG) performance status of 2 due to profound fatigue and anaemia. The examination was largely unremarkable apart from marked conjunctival pallor. Focused abdominal examination revealed tenderness to deep palpation in the epigastric region; however, the abdomen remained soft and non-distended with no evidence of guarding, rebound, or organomegaly. No peripheral oedema or lymphadenopathy was identified.

Initial abdominal ultrasonography revealed a 9-cm mass located on the lesser curvature of the stomach, with evidence of local invasion into the adjacent liver and pancreas. To further assess the extent of the disease, a whole-body positron emission tomography–computed tomography (PET–CT) scan with intravenous F-18 fluorodeoxyglucose (FDG) was performed. This scan included a 16-slice contrast-enhanced CT for attenuation correction and anatomical co-registration.

PET–CT demonstrated a hypermetabolic gastric mass measuring 9 × 11 cm (SUVmax 10.3). The lesion was centred on the lesser curvature, extended into the gastric antrum, and infiltrated adjacent mesenteric fat planes. Possible invasion of the left hepatic lobe was also noted. Multiple mildly FDG-avid perigastric lymph nodes were observed (largest measuring 1.8 × 1.8 cm, SUVmax = 2), located anterior to the head of the pancreas. Hepatic uptake was physiological, with no focal lesions identified. Splenic activity was within normal limits, and no FDG-avid para-aortic, pelvic, or inguinal lymphadenopathy was present.

**Fig. 1:**
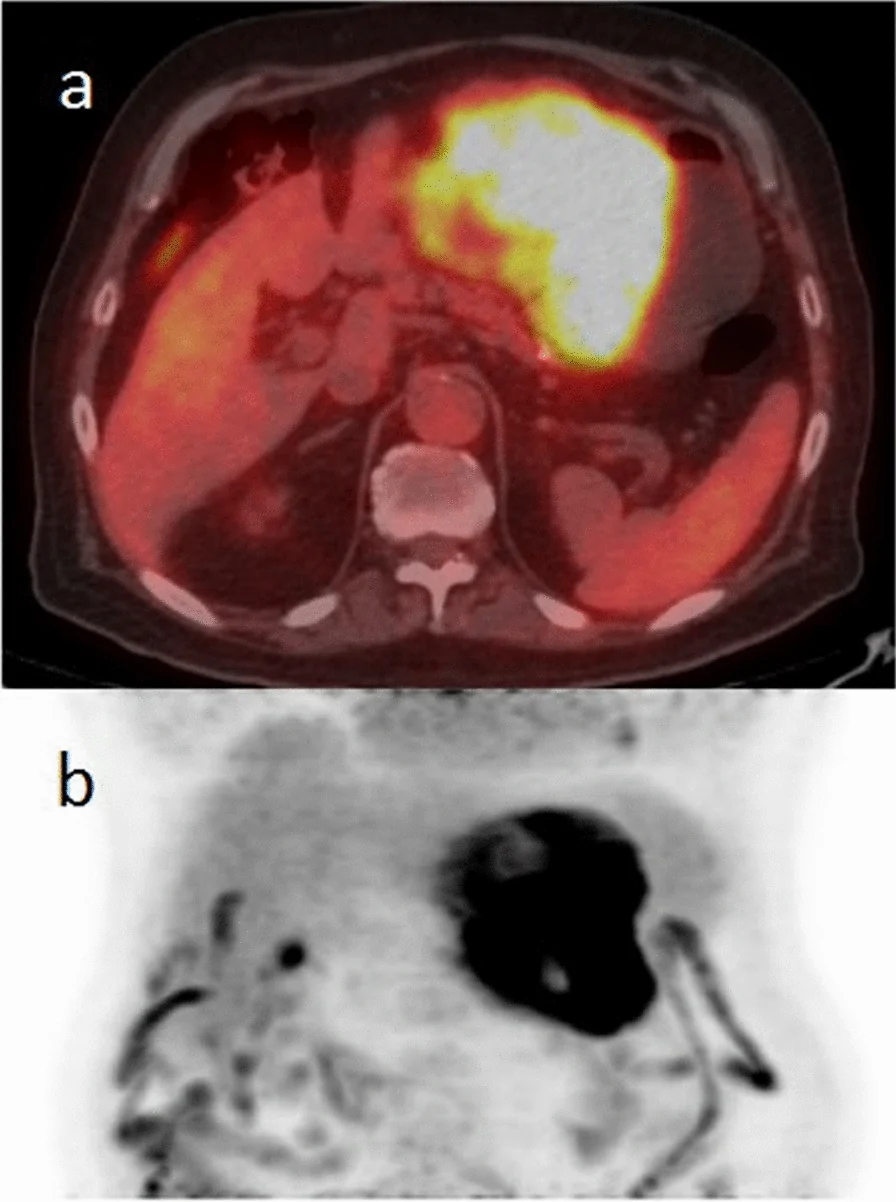
Positron emission tomography–computed tomography images of the gastric lesion. **a** Axial positron emission tomography–computed tomography demonstrating a hypermetabolic mass along the lesser curvature of the stomach. **b** Maximum intensity projection in 3D view revealing an irregular, FDG-avid mass in the same location

Findings were consistent with an active gastric neoplasm with invasion of adjacent organs, regional lymph node involvement and no evidence of distant metastatic disease (Fig. 1). Based on these findings, the tumour was classified as stage IVA (cT4bN + M0) according to the American Joint Committee in Cancer (AJCC) 8th Edition cTNM clinical staging system.

**Fig. 2:**
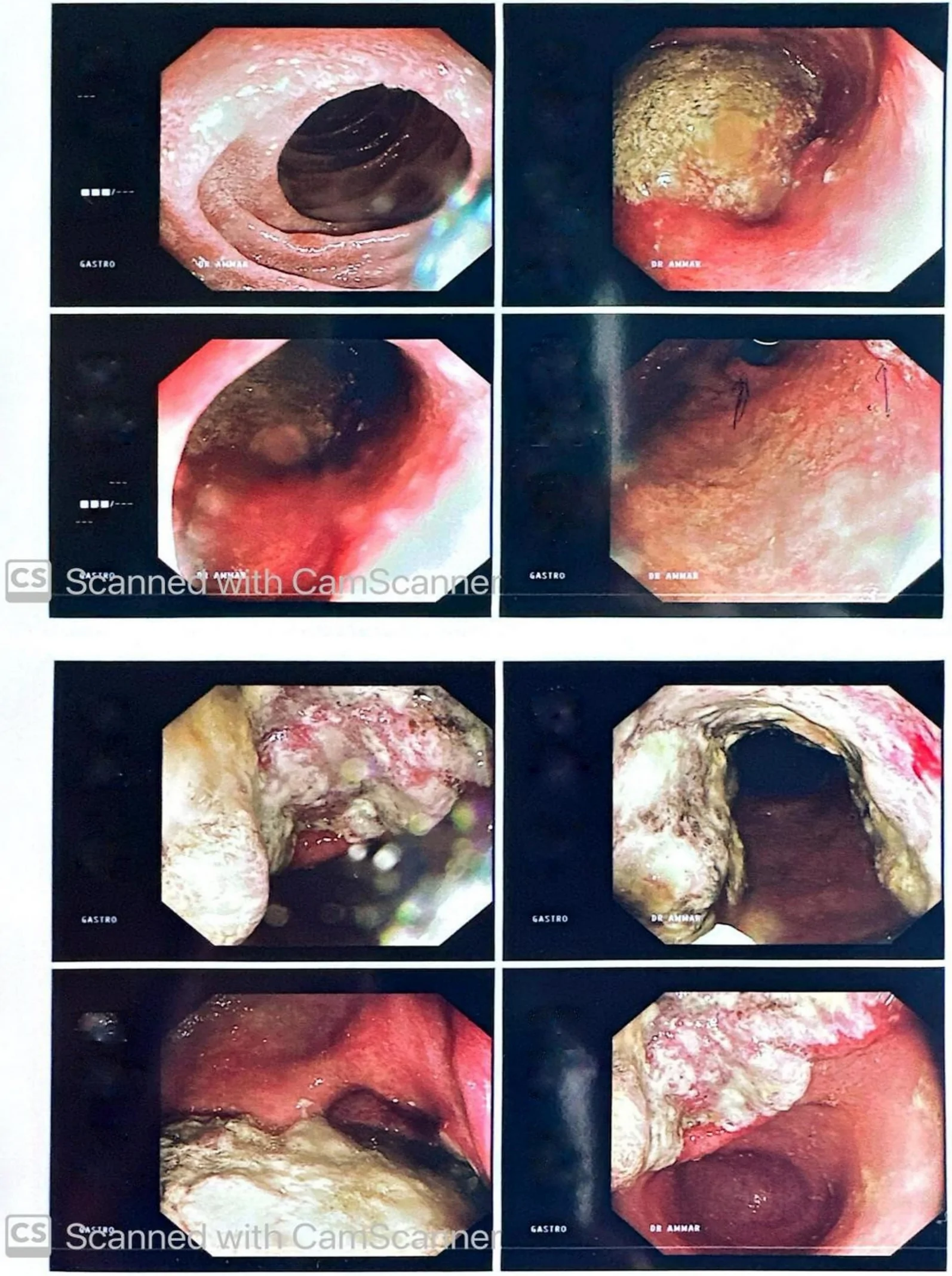
Endoscopic findings

Subsequently, an upper gastrointestinal endoscopy was performed using an Olympus upper endoscope. The endoscope was advanced to the second portion of the duodenum. The oesophagus and gastroesophageal junction appeared normal. Moderate to large residual food contents were noted within the gastric body. A large, deeply ulcerated, friable mass was identified along the lesser curvature of the gastric body. The lesion began 4 cm distal to the GE junction, extended through the incisura into the proximal antrum and ended approximately 3 cm proximal to the pylorus. The mass occupied approximately 70% of the luminal circumference. Retroflexion confirmed the mass on the lesser curvature. The duodenal bulb and second part of the duodenum appeared normal. Multiple biopsies were obtained, with minor bleeding that resolved spontaneously (Fig. 2).

**Fig. 3:**
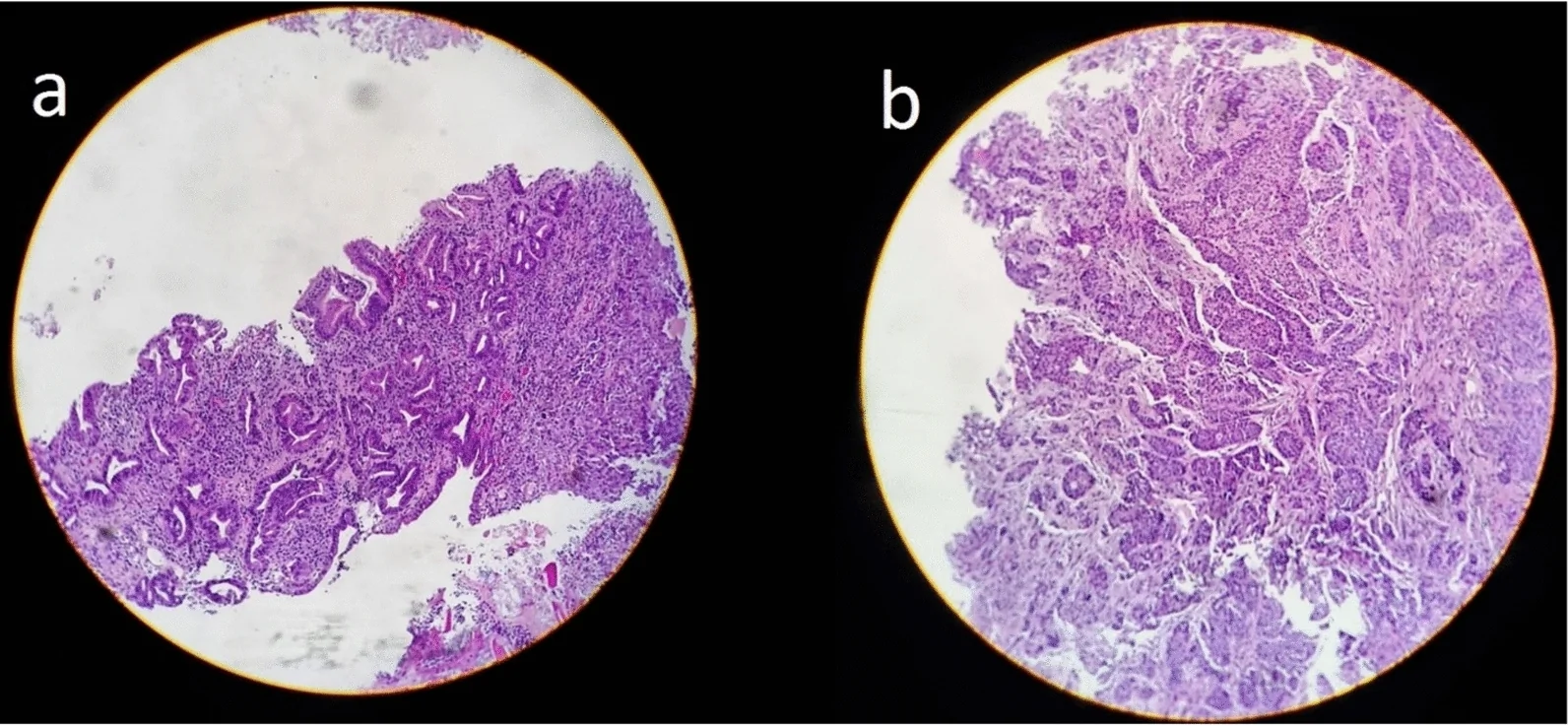
Microscopic view of the gastric biopsy

Histopathological examination of the gastric biopsy revealed a case of poorly differentiated adenocarcinoma with squamous differentiation (Fig. 3).

**Fig. 4:**
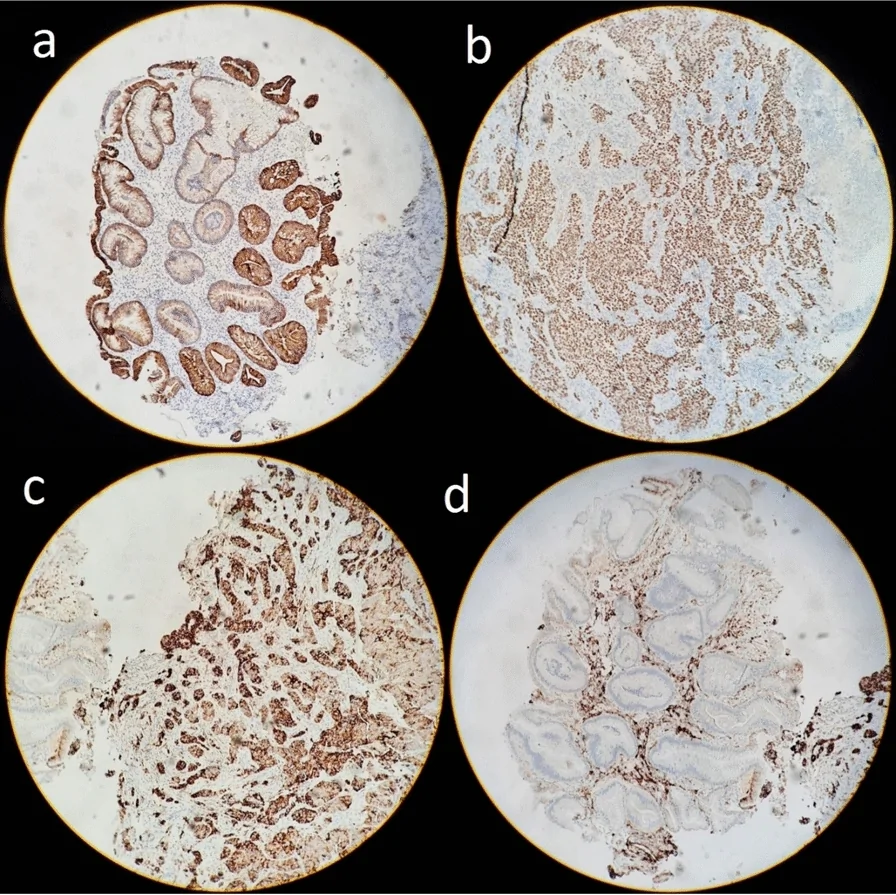
Immunohistochemical staining of the biopsy revealing a focal positivity for cytokeratin 7, diffuse positivity for p40 and programmed death-ligand 1 positivity (**a** cytokeratin 7, **b** protein 40, **c**, **d** programmed death-ligand 1)

Immunohistochemistry demonstrated tumour cells diffusely positive for p40 and focally positive for cytokeratin 7 (CK7). The tumour was negative for CDX2, CK20, and synaptophysin. Programmed death-ligand 1 (PD-L1) expression was evaluated using the automated DAKO 22C3 PharmDx immunohistochemistry assay, revealing a combined positive score (CPS) of 75 (Fig. 4).

In view of the patient's baseline performance status (ECOG 2), advanced age, associated comorbidities, and anticipated high operative risk, a curative-intent strategy comprising induction chemotherapy combined with immunotherapy was undertaken, followed by planned reassessment for surgical resection based on treatment response. This non-surgical approach was further strongly supported by the tumour’s high PD-L1 expression (CPS 75), which identified the patient as an ideal candidate for immune checkpoint inhibition and suggested a high probability of systemic treatment sensitivity.

The patient was started on a combined chemotherapy and immunotherapy regimen beginning on July 10, 2024. The treatment plan included:

- Chemotherapy: modified FOLFOX6 (mFOLFOX6) regimen (oxaliplatin, 5-fluorouracil and leucovorin), administered every two weeks for a total of 12 cycles (IV infusion over 48 h).
- Immunotherapy: Nivolumab, administered every two weeks for 12 cycles

**Fig. 5:**
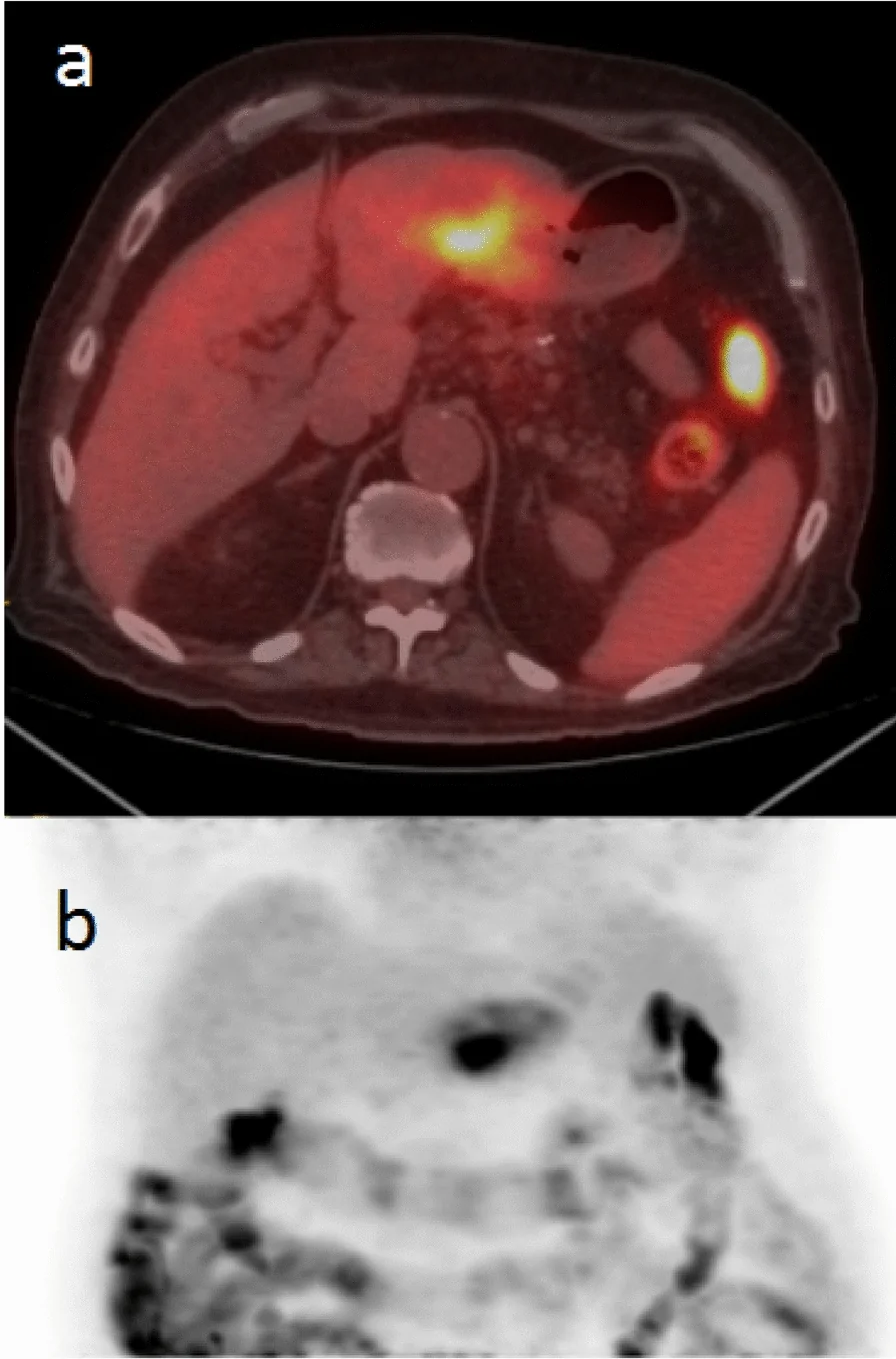
Reduction in tumour size at 3-month follow-up

Follow-up imaging and response assessment:

On October 16, 2024, the patient underwent a follow-up whole-body PET–CT scan to assess the response to treatment for gastric carcinoma. When compared to the initial PET–CT performed on July 9, 2024, the scan demonstrated:

- A significant reduction in the size and metabolic activity of the primary gastric mass. The lesion, previously measuring 11 × 9 cm, had decreased to 7 × 5 cm in maximal dimensions. The maximum standardized uptake value (SUVmax) also declined from 10.3 to 7.0. The mass remained located at the level of the gastric antrum, with moderate residual infiltration into the adjacent mesenteric fat planes and the left hepatic lobe (Fig. 5).
- A notable reduction in the size and FDG avidity of the perigastric lymph nodes. The largest node measured 1.0 cm in short-axis diameter (previously 1.8 cm), with a decrease in SUVmax from 2.0 to 1.6.

These findings were consistent with a partial response (PR) according to the Response Evaluation Criteria in Solid Tumours (RECIST 1.1), based on a 36.4% reduction in the maximal longitudinal diameter of the primary lesion. Metabolically, the tumour concurrently exhibited a PR according to the PET Response Criteria in solid tumours (PERCIST 1.0) based on 32.0% reduction in its SUVmax (Fig. 5).

Subsequently, on January 12, 2025, the patient underwent a contrast-enhanced CT scan of the abdomen. The imaging showed no evidence of mucosal thickening in the gastric wall, suggesting resolution of the previously visualized mass and further supporting a favourable response to treatment.

**Fig. 6:**
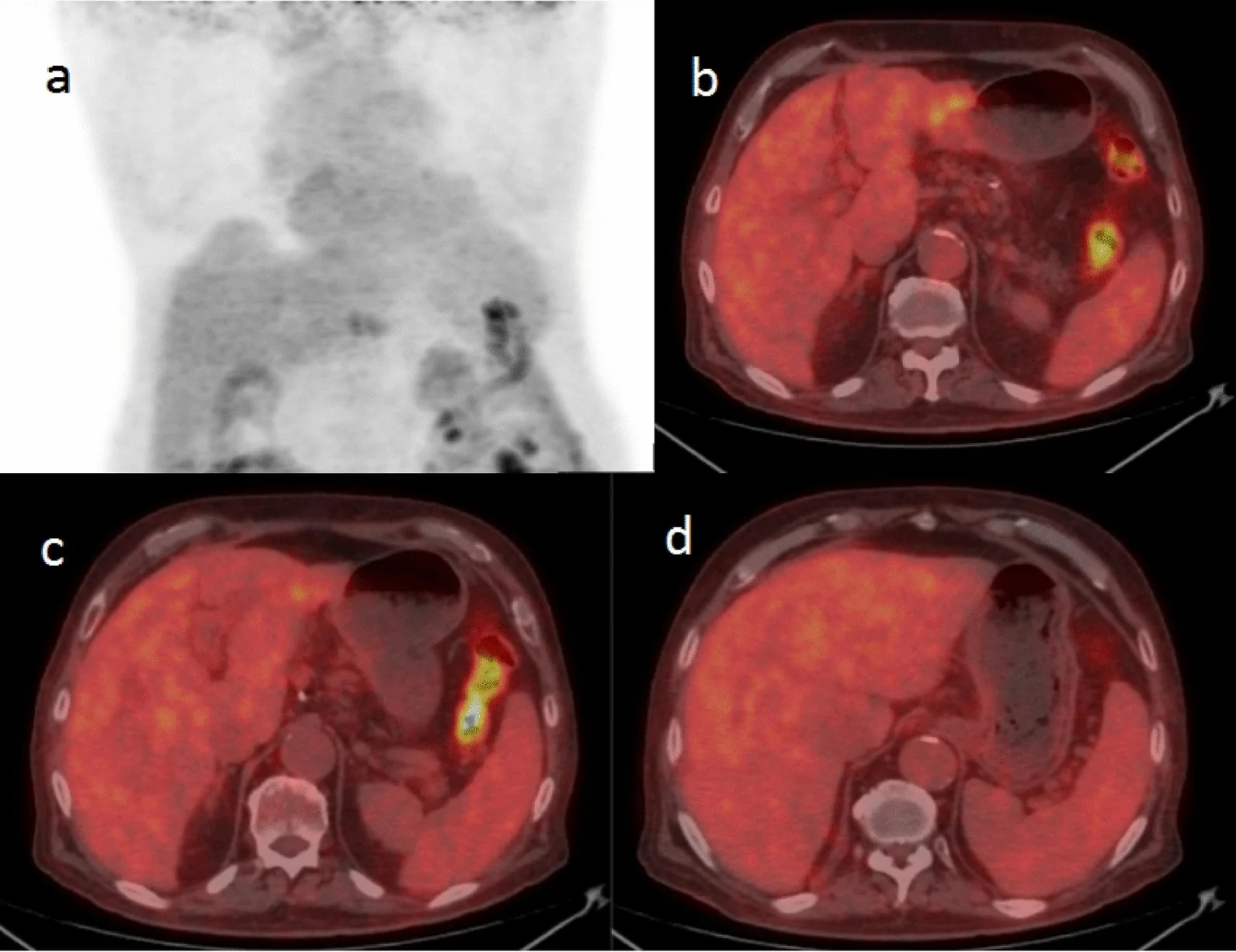
Positron emission tomography–computed tomography scan at 1-year follow-up demonstrating complete metabolic response

At the most recent follow-up on June 24, 2025, a PET scan demonstrated no evidence of residual or recurrent disease as shown in Fig. 6.

Collectively, these findings confirmed a durable Radiologic and Metabolic Complete response (CR) according to RECIST 1.1 and PERCIST 1.0 criteria.

Regarding treatment tolerability and adverse effects:

Throughout the entire course of treatment, the patient reported no pain or discomfort related to the therapeutic regimen.

The patient experienced treatment-related myelosuppression; symptomatic anaemia was effectively managed with red blood cell transfusions, while neutropenia was addressed with subcutaneous injections of filgrastim, a granulocyte colony-stimulating factor (G-CSF), to stimulate leukocyte production and maintain immune function.

Nausea and vomiting were well-controlled with antiemetic therapy, and did not interfere significantly with quality of life. On occasion, the patient experienced transient episodes of diarrhea, xerostomia (dry mouth), and hypotension, all of which were managed symptomatically and did not necessitate interruption of treatment.

Following tumour regression, the patient was commenced on maintenance therapy with nivolumab in combination with oral capecitabine, planned for a duration of 1 year to minimize the risk of recurrence and consolidate the initial therapeutic response.

**Fig. 7:**
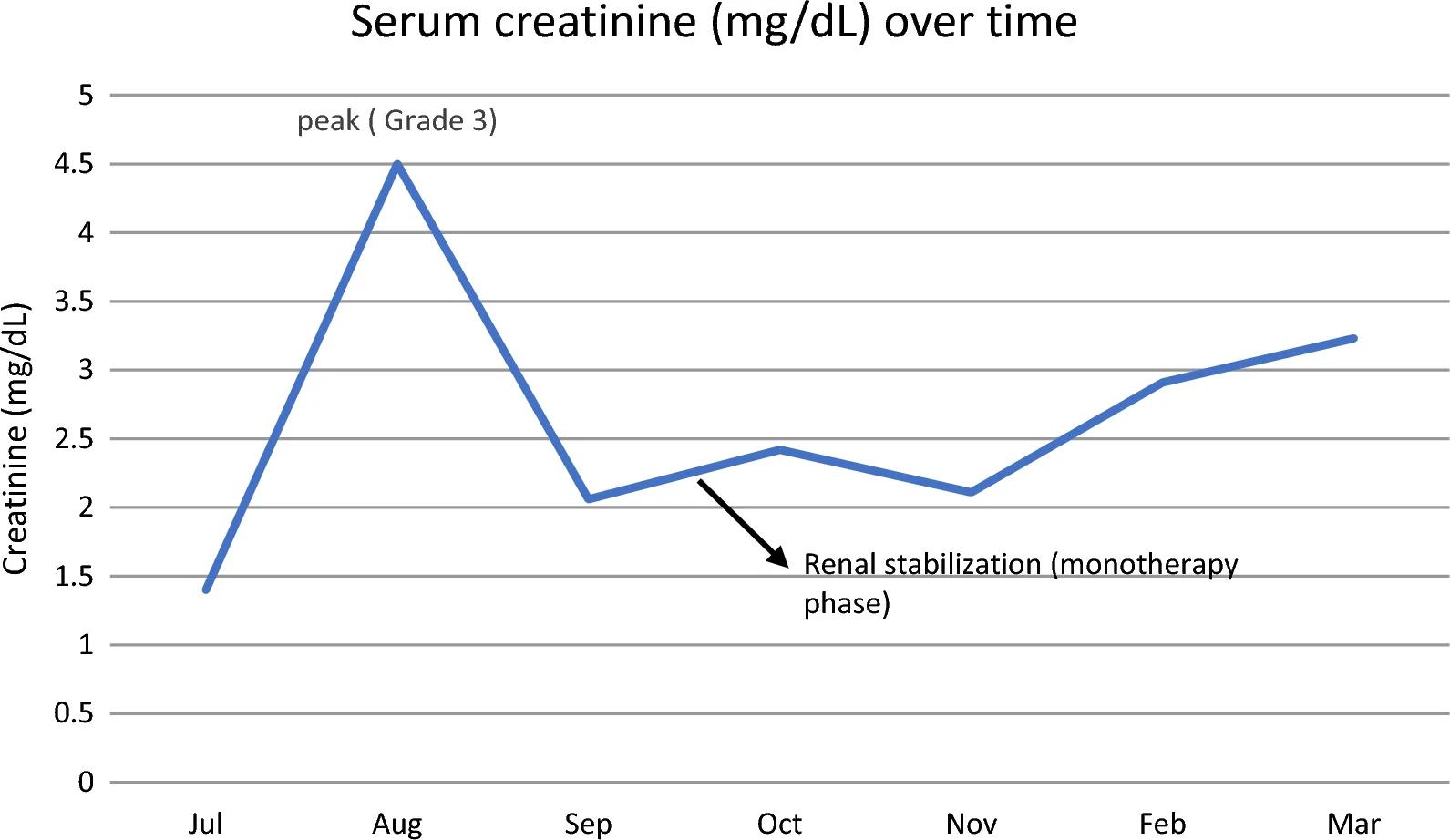
Serum creatinine levels (July 2025–March 2026)

During the maintenance period, the patient developed multiple severe episodes of gastroenteritis, which were initially managed conservatively with oral antibiotics and sporadic intravenous hydration. However, in August 2025, the compounding gastrointestinal fluid losses precipitated a severe Grade 3 acute kidney injury (AKI), leading to profound decompensated metabolic acidosis. On emergency presentation, the patient manifested severe vomiting, lethargy, and altered consciousness. Urgent laboratory investigations revealed a critical peak serum creatinine (SCr) of 4.50 mg/dL (elevated from a baseline of 0.90 mg/dL), a blood urea nitrogen (BUN) of 84 mg/dL, and an arterial blood gas demonstrating an advanced metabolic acidosis with a pH level of 7.268. Diagnostic evaluation initiated at the time of injury included a comprehensive urinalysis, which revealed an elevated urine specific gravity (1.028) and a fractional excretion of sodium (FeNa) of < 1%, heavily favouring a pre-renal aetiology; notably, active urinary sediments, haematuria, and significant proteinuria were entirely absent. Both capecitabine and nivolumab were immediately withheld during this acute phase, and an urgent nephrology consultation was obtained. Comprehensive supportive measures were instituted immediately, including aggressive fluid resuscitation with intravenous isotonic saline, continuous telemetry monitoring, and the administration of intravenous sodium bicarbonate infusions to correct the life-threatening acidemia. Following these intensive supportive interventions alone, the patient’s clinical status and renal function demonstrated rapid, steady improvement without the need for renal replacement therapy. While the severity of the initial ischemic and toxic insult resulted in structural parenchymal damage and a subsequent transition to stage 3 chronic kidney disease (CKD), the SCr successfully stabilized within a new baseline range of 2.0 to 2.6 mg/dL. Figure 7 illustrates the longitudinal trend of serum creatinine levels, beginning with the initiation of maintenance therapy in July 2025 until the most recent follow-up in March 2026.

A systematic summary of these treatment-related toxicities, graded according to the Common Terminology Criteria for Adverse events (CTCAE) v5.0, is presented in Table 1.

**Table 1:** Adverse event profile

Adverse event	CTCAE v5.0 Grade
Acute kidney injury	Grade 3
Symptomatic anaemia	Grade 2
Neutropenia	Grade 2
Gastroenteritis	Grade 2
Nausea and vomiting	Grade 1
Diarrhea	Grade 1
Xerostomia	Grade 1
Hypotension	Grade 1

*CTCAE* common terminology criteria for adverse events

At the time of writing, the patient remains clinically well with no evidence of disease recurrence. Notably, no surgical intervention was required, and clinical complete remission was achieved with systemic chemotherapy and immunotherapy alone. The patient is currently continuing nivolumab monotherapy as part of a planned 1-year course, with ongoing follow-up, including clinical assessment and serial measurements of tumour markers (CEA, CA 19-9 and CA 125). Carcinoembryonic Antigen (CEA) and cancer antigen (CA 125) remained within the normal limits throughout the disease course. In contrast, carbohydrate antigen 19-9 (CA 19-9) was persistently elevated but demonstrated a gradual decline over time (93.11 U/mL in May 2025, 62.54 U/mL in October 2025 and 53.18 U/mL in February 2026), consistent with a favourable biochemical trend.

Upon completion of the treatment period, a positron emission tomography–computed tomography (PET–CT) scan is planned to evaluate treatment response and confirm disease status. This non-invasive surveillance strategy was deliberately chosen over follow-up upper endoscopy and repeat tissue biopsies to avoid unnecessary bleeding, sedation and procedural risks given the patient’s advanced age, associated cardiovascular comorbidities, and severe renal impairment. Long-term outcomes remain to be determined, as follow-up is ongoing.

A summary of the patient’s clinical course and key findings is presented in Table 2.

**Table 2:** Timeline of diagnosis, treatment, and follow-up of the patient

Date	Event	Key findings
July 9, 2024	Initial PET–CT	11 × 9 cm hypermetabolic gastric mass with perigastric lymphadenopathy
July 10, 2024	Treatment initiated (oxaliplatin + 5-FU + Nivolumab)	Start of combined chemo-immunotherapy
October 16, 2024	3-month follow-up PET–CT	Tumour reduced to 7 × 5 cm; SUVmax **↓** from 10.3 to 7.0
January 12, 2025	Contrast enhanced CT	No visible mucosal thickening; near complete radiologic resolution
June 24, 2025	PET–CT	Complete metabolic response; no recurrence
July 2025–present	Maintenance therapy for a period of 1 year	Patient remains disease free with good quality of life

*PET–CT* Positron emission tomography–computed tomography, *5-FU* 5-Fluorouracil,* SUVmax* maximum standardized uptake value

## Discussion

This case presented a rare instance of poorly differentiated gastric adenocarcinoma with squamous differentiation in a 75-year-old male. The patient initially presented with nonspecific symptoms, including anaemia, weight loss, and anorexia. Notably, the diagnosis followed a misleading clinical course, where the initial symptoms were first evaluated in the context of prostatitis, underlining the importance of persistent investigation in elderly patients with unexplained anaemia.

A significant diagnostic challenge in this case was the histological classification. According to the Japanese Gastric Cancer Association and the WHO criteria, a diagnosis of adenosquamous carcinoma requires the squamous component to comprise at least 25% of the total tumour volume [3, 4]. In our patient, while the initial biopsy demonstrated diffuse p40 positivity—a highly specific marker for squamous lineage [8, 9], the focal nature of the CK7 staining and the lack of a full surgical specimen necessitated the more conservative diagnosis of ‘poorly differentiated adenocarcinoma with squamous differentiation.’ However, the clinical behaviour and the metabolic intensity on PET–CT (SUVmax 10.3) were consistent with the highly aggressive nature typically observed in true ASC, which is often associated with early lymphatic involvement and direct hepatic invasion [10].

The most remarkable clinical feature of this case was the exceptionally high PD-L1 expression, demonstrated by a CPS of 75. In gastric cancer, the CPS is the standard biomarker used to predict the efficacy of PD-L1 inhibitors like nivolumab [11]. The landmark CheckMate 649 trial established that in the first-line advanced or metastatic setting, patients with a PD-L1 CPS ≥ 5 derived a significant survival benefit from adding nivolumab to standard chemotherapy, achieving an objective response rate (ORR) of approximately 60% and a complete response (CR) rate of 12 to 13% in high expressors [6]. Although CheckMate 649 targeted metastatic disease, our patient's locally advanced (T4b) tumour displayed a CPS of 75, representing a "super-responder" status that far exceeds the standard thresholds, likely driving the durable non-surgical remission. This remarkable response stems from the synergism between cytotoxic chemotherapy and PD-1 inhibition. Oxaliplatin and 5-fluorouracil are known to induce immunogenic cell death, which releases tumour-associated antigens and pro-inflammatory cytokines. This process effectively primes the tumour microenvironment, enhancing the visibility of malignant cells to the immune system and amplifying the efficacy of nivolumab, particularly in a high-CPS environment [12]. Interestingly, while pure gastric adenocarcinomas typically exhibit variable PD-L1 expression, tumours with squamous differentiation—often marked by diffuse p40 positivity—may be associated with a more ‘inflamed’ and immune-active tumour microenvironment [13].

A potential limitation of this case is the lack of Microsatellite Instability (MSI) or Mismatch Repair (MMR) testing, which are established biomarkers for immunotherapy sensitivity. MSI–high tumours frequently exhibit high PD-L1 expression and dramatic responses to PD-1 blockade. However, large-scale trials such as CheckMate 649 have shown that CPS is an independent predictor of benefit from nivolumab, regardless of MSI status [6].

The metabolic intensity of the primary tumour (SUVmax 10.3) in our patient further aligns with recent evidence suggesting that high 18F-FDG uptake on PET–CT correlates significantly with positive PD-L1 status in gastric malignancies [14]. This suggests that the aggressive squamous features, while historically linked to poor prognosis, may have paradoxically served as a marker for extreme sensitivity to immune checkpoint blockade. This patient’s super-responder status (CPS 75) likely drove the transition from a locally invasive T4b mass to a durable radiological complete response.

The management of this 75-year-old patient illustrates the evolving paradigm of personalized oncology, where comprehensive histopathological and molecular profiling informs highly individualized treatment planning. Initially, the 11-cm mass’s extensive infiltration into the mesenteric fat planes, pancreas, and left hepatic lobe rendered a microscopically margin-negative (R0) resection highly improbable and surgically morbid. Given the patient’s advanced age, baseline systemic symptoms, and comorbidities, an upfront total gastrectomy with D2 lymphadenectomy carried prohibitive perioperative risks. Consequently, a tailored "chemo-immunotherapy first" strategy was adopted to assess tumour biology and optimize the patient’s clinical status. This immune-signature-directed approach was strongly validated by the patient’s combined positive score (CPS) of 75. While standard guidelines for locally advanced gastric cancer remain firmly surgery-based whenever feasible, the subsequent total resolution of mucosal thickening on CT and a complete metabolic response on PET shifted the clinical calculation for this specific individual. In this unique context, the incremental oncological benefit of a salvage gastrectomy was heavily outweighed by the immediate risk of severe postoperative complications, such as malabsorption and dumping syndrome, which would have severely compromised the patient’s quality of life. This highlights how biomarker-guided profiling can guide safe, non-surgical management pathways in carefully selected patients who face unacceptable surgical risks.

The clinical course was acutely compromised by Grade 3 Acute Kidney Injury (AKI), defined by a peak serum creatinine (SCr) of 4.50 mg/dL in August 2025. According to the KDIGO (kidney disease: improving global outcomes) criteria [15], this represents a severe, life-threatening insult, further evidenced by the patient’s profound metabolic acidosis (pH 7.268). At this juncture, the diagnostic challenge was to distinguish between three overlapping aetiologies: pre-renal azotaemia from dehydration, direct tubular toxicity from capecitabine [16] further exacerbated by the patient’s advanced age [17], and potential immune-mediated nephritis from nivolumab [18].

To resolve this diagnostic ambiguity, a strict clinical and biochemical exclusion framework was utilized. Immune-related nephritis typically presents with an insidious onset, often accompanied by pyuria, haematuria, or sub-nephrotic range proteinuria, and fundamentally fails to improve with volume expansion alone. In our patient, the complete absence of active urinary sediments, the low fractional excretion of sodium (FeNa < 1%), and the immediate, dramatic turnaround of serum creatinine following aggressive fluid resuscitation strongly pointed toward a combination of profound pre-renal hypovolemia and acute tubular injury from capecitabine. Consequently, systemic corticosteroids were deliberately withheld. The clinical rationale was clear: initiating high-dose steroids was unnecessary given the prompt response to supportive hydration alone, and introducing immunosuppression would have carried an unfavourable risk profile by potentially blunting the patient's highly beneficial anti-tumour immune response driven by his high PD-L1 expression (CPS 75).

Given the diagnostic data, a "multifactorial subtraction strategy" was employed. This approach prioritized the removal of the most likely reversible drivers of the 4.50 mg/dL peak—specifically the cytotoxic chemotherapy and the associated gastrointestinal distress (vomiting) that precipitated hypovolemia. Discontinuing the renally cleared capecitabine was necessary to halt direct tubular insult, while the decision to safely resume nivolumab was justified by the patient's high PD-L1 expression (CPS 75) [6] and the significant risk of oncologic recurrence.

The long-term outcome of this strategy firmly validated our clinical reasoning and biochemical exclusion. Following the complete withdrawal of chemotherapy and the continuation of nivolumab monotherapy, the SCr remained highly stable within its new baseline range between 2.0 and 2.6 mg/dL (e.g., 2.06 in September; 2.11 in November) as shown in Fig. 7. The complete absence of secondary acute creatinine "spikes" or progressive renal degradation over months of ongoing immune checkpoint inhibition definitively confirms that immunotherapy was not the driver of the initial renal collapse. This maintenance of renal stability allowed for the successful continuation of life-saving immunotherapy in a high responder without further acute degradation. The patient is currently receiving pharmacological management for chronic kidney disease to manage blood pressure and metabolic balance. Despite the persistent elevation in serum creatinine, the patient reports a high quality of life, remains in good clinical status and continues to tolerate the immunotherapy without further renal or gastrointestinal complications.

While the patient achieved a complete radiological response, the CA 19-9 demonstrated a protracted biochemical washout, declining from 93.11 to 53.18 U/mL over the maintenance phase, representing a cumulative 42.9% reduction. In the absence of biliary or inflammatory biology, this persistent elevation likely reflects a biological lag rather than residual macro-disease. Such kinetic trends, where the trajectory of the marker is downward, are often more clinically significant than the absolute reference threshold [19]. A meta-analysis of over 11,000 patients confirmed that CA 19-9 levels correlate significantly with tumour stage and overall survival [19]. Furthermore, changes in CA 19-9, specifically shifts exceeding 20%, have been identified as independent risk factors for survival, particularly in poorly differentiated subtypes [20]. In this instance, the 40% decline in CA 19-9 served as the sole informative biomarker, providing biochemical validation for the continued use of nivolumab monotherapy as a consolidated maintenance strategy in this aggressive, poorly differentiated subtype. In the context of a complete metabolic response (SUVmax resolution) and a declining CA 19-9 trend, the multidisciplinary team decided to defer invasive endoscopic procedures given the patient’s age and the definitive serial radiological resolution of the 11-cm mass and mucosal thickening on PET–CT and contrast-enhanced CT.

Tumour cells in this case were focally positive for CK7, a low-molecular-weight intermediate filament typically found in the ductal and glandular epithelium of the proximal gastrointestinal tract [21]. While CK7 is absent in normal gastric mucosa, its expression is often induced by chronic mucosal irritation and is present in approximately 50 to 70% of gastric adenocarcinoma [21]. The prognostic value of CK7 in gastric carcinoma remains controversial. A study published in 2021 reported no significant association between CK7 expression and patient outcomes [21]. Conversely, another investigation demonstrated that CK7 expression was correlated with larger tumour size and poor differentiation, both well-recognized indicators of unfavourable prognosis [22]. These conflicting findings suggest that the role of CK7 as a prognostic biomarker remains uncertain and requires validation in larger, multicentre studies. In this patient, the high PD-L1 (CPS) expression likely served as a more robust predictor of both prognosis and treatment response than the focal CK7 status.

Contextualizing this case within the global literature highlights the unique therapeutic landscape of rare gastric cancer variants and helps frame our patient's outcome against experiences from other centres. Primary gastric malignancies presenting with squamous components—whether classified as pure primary gastric squamous cell carcinoma (GSCC) or adenosquamous carcinoma (GASC)—are historically linked to a highly aggressive clinical course and dismal responses to conventional chemotherapy. However, emerging clinical evidence suggests these variants may possess a distinct, highly immune-active tumour microenvironment. For instance, a recent case report evaluating advanced metastatic gastric squamous carcinoma documented a remarkable long-term survival response exceeding 49 months using an integrated nivolumab-based regimen [13]. Similarly, another report detailed a 33-year-old male with highly invasive, poorly differentiated stage IV GSCC who was treated with a combination of leucovorin, fluorouracil, oxaliplatin (FOLFOX), and nivolumab, illustrating the complex management and acute toxicities often tied to these aggressive phenotypes [23]. Crucially, broader institutional immunoprofiling of gastric tumours with squamous differentiation confirms that these rare variants yield significantly higher rates of positive combined positive scores (CPS). This is exemplified by a reported stage IV GASC patient with a high CPS (≥ 10) who achieved a durable 14-month progression-free survival utilizing frontline nivolumab monotherapy [9]. Much like our 75-year-old patient, these parallel cases demonstrate that tumours exhibiting squamous differentiation can display severe sensitivity to immune checkpoint blockade. By validating our observations against these global experiences, it becomes evident that looking beyond traditional staging to embrace comprehensive molecular tracking is vital to uncovering these specific "super-responders."

An important consideration highlighted by this case is the limited accessibility to advanced oncologic care in resource-constrained settings, such as Syria [24]. This includes not only restricted availability of immune checkpoint inhibitors, such as nivolumab, due to geopolitical barriers and economical constraints, but also limited access to early detection strategies and advanced molecular and immunohistochemical analysis required to guide personalized therapy (24). In particular, PD-L1 testing, despite its critical role in predicting the response to immunotherapy, is not routinely performed in all patients, potentially leading to missed opportunities for optimal treatment selection. The high cost of immunotherapy further restricts its use compared to conventional chemotherapy, rendering it inaccessible to a large proportion of patients.

Although our patient was ultimately able to receive appropriate treatment by travelling to Lebanon. Frequent cross-border travel came with a significant logistical and physical burden, posing a substantial challenge especially given his advanced age and initial systemic frailty. This underscores the critical importance of ensuring availability of advanced diagnostic tools and therapies within patients’ home countries.

## Conclusion

This case demonstrates the vital importance of comprehensive histopathological profiling, immune-signature-directed therapy, and individualized treatment planning in aggressive gastric cancer subtypes. While the disease displayed highly aggressive features—including poor differentiation, a squamous component, and an invasive nature—the presence of an exceptionally high PD-L1 expression (CPS 75) served as a powerful guide for tailored therapeutic intervention. The clinical course suggests that squamous differentiation, high FDG uptake, and high PD-L1 status may collectively signal a more immune-active tumour environment, rendering the malignancy exceptionally susceptible to checkpoint inhibition. Rather than establishing a broad organ-preserving standard, this case underscores the value of personalized oncology; carefully selected patients with favourable molecular signatures and high surgical risks may achieve durable clinical and metabolic responses through biomarker-guided strategies. While prolonged longitudinal follow-up remains essential to evaluate the long-term durability of this non-surgical approach, this experience supports the use of detailed molecular tracking to optimize patient outcomes while preserving functional quality of life.

## Data Availability

No datasets were generated or analysed during the current study.

## Abbreviations

AJCC: American Joint Committee on Cancer
AKI: Acute kidney injury
FeNa: Fractional excretion of sodium
KDIGO: Kidney disease: improving global outcomes
SCr: Serum creatinine
mFOLFOX6: Modified Fluorouracil, Leucovorin and Oxaliplatin
MSI: Microsatellite instability
MMR: Mismatch repair
ORR: Objective response rate
CR: Complete response
PR: Partial response
ASC: Adenosquamous carcinoma
CA 125: Cancer antigen 125
CEA: Carcinoembryonic antigen
GAC: Gastric adenocarcinoma
CA 19-9: Carbohydrate antigen 19-9
p40: Protein 40
CDX2: Caudal Type Homeobox 2
CK7: Cytokeratin 7
CK20: Cytokeratin 20
CPS: Combined positive score
CT: Computed tomography
FDG: Fluorodeoxyglucose
5-FU: 5-Fluorouracil
GE junction: Gastroesophageal junction
G-CSF: Granulocyte colony-stimulating factor
IHC: Immunohistochemistry
IV: Intravenous
PD-1: Programmed cell death protein 1
PD-L1: Programmed death-ligand 1
PET–CT: Positron emission tomography–computed tomography
PSA: Prostate-specific antigen
SUVmax: Maximum Standardized Uptake Value
TMN: Tumour, node, metastasis
WHO: World Health Organization
CTCAE: Common Terminology Criteria for Adverse Events
PERCIST: Positron Emission Tomography Response Criteria
RECIST: Response Evaluation Criteria in Solid Tumours
ECOG: Eastern Cooperative Oncology Group
